# Highly efficient thermal deformation optimization method for smart-cut mirrors over the entire photon energy range

**DOI:** 10.1107/S1600577522007160

**Published:** 2022-07-29

**Authors:** Shaofeng Wang, Dongni Zhang, Ming Li, Lidan Gao, Minwei Chen, Fugui Yang, Weifan Sheng

**Affiliations:** a Institute of High Energy Physics, Chinese Academy of Science, Beijing 100049, People’s Republic of China; b University of Chinese Academy of Sciences, Beijing 100049, People’s Republic of China; RIKEN SPring-8 Center, Japan

**Keywords:** thermal deformation optimization, white-beam mirror, entire photon energy range, finite-element analysis

## Abstract

A method to optimize the notches of water-cooled white-beam mirrors over the entire photon energy range is proposed. A theoretical method is used to quantitatively evaluate the influence of the thermal load on the thermal deformation of a mirror. The result of theoretical calculations and finite-element analysis are consistent, which proves the feasibility of the method. The root mean square of the curvatures of the thermal deformation of the white-beam mirror over the entire photon energy range can be minimized. This method greatly simplifies the design work of water-cooled white-beam mirrors.

## Abstract 2

A theoretical method is proposed to optimize the notches of water-cooled white-beam mirrors over the entire photon energy range.

## 1. Introduction

The High Energy Photon Source (HEPS) is a diffraction-limited storage ring synchrotron radiation source with better than 60 pm rad emittance and 6 GeV electron energy, which will be completed in 2025 in Huairou, Beijing, China (Li *et al.*, 2021 ▸). Fifteen beamlines are under construction during the first phase. As the first optical beamline component, the white-beam mirror (WBM) is usually designed to absorb excess heat load and needs to be cooled. In the field of synchrotron radiation, cooling methods include direct (Lee *et al.*, 2001 ▸; Liu *et al.*, 2014 ▸) and indirect cooling, and the cooling fluids include gas (Toellner *et al.*, 2006 ▸), water and liquid nitro­gen (Cutler *et al.*, 2020 ▸; Stimson *et al.*, 2019 ▸). For WBMs, for reasons including thermal power, cost and sealing, indirect water cooling is widely adopted. The thermal deformation of a WBM can be simplified as the bending deformation caused by the temperature gradient along the mirror thickness direction and the bump deformation caused by the temperature gradient due to uneven heat flow along the mirror length direction. In order to reduce thermal deformation and improve reflection quality, Zhang *et al.* (2013 ▸) proposed cooling the mirror along the top side of the substrate and optimized the temperature distribution of the mirror section with notches (‘smart cuts’). This method can completely eliminate the bending deformation in the meridional direction. When the power distribution is not uniform, there will be a superposition of bending and bump deformation. Suppressing part of the bump deformation by the bending deformation is still a desire when designing the structure of a WBM. Furthermore, the heat load of the WBM is not fixed when changing the beamline energy by adjusting the gap of the undulator, which means both the total power and distribution of power density will change. In this case, finding a fixed average state structure is a problem in the design of a WBM.

For a given working condition, as the depth of notches (*D*) increases, the thermal deformation slope error first decreases and then increases. The value of *D* corresponding to the smallest slope error is the optimal notches depth (*D*_opt_) under this working condition. The average value of *D*_opt_ over the entire photon energy range can be regarded as the optimal notch depth over the entire photon energy range (*D*_opt-all_) (Rudolf *et al.*, 2014 ▸), but the workload is also quite large. This study proposes a highly efficient optimization method for ‘smart cuts’ based on theoretical analysis for removing circle deformation which leads to a de-focusing effect. Only one round of optimization is needed to find *D*_opt-all_. The worst condition analyzed by the theoretical method is used to determine whether the WBM meets our optical requirements over the entire energy range.

## 2. Theoretical analysis

The basic idea of WBM optimization is to find a suitable notch depth so that the bending deformation of the mirror in the meridian direction can offset the bump deformation as much as possible. In this study, we only care about the thermal deformation in the meridional direction, so the theoretical analysis shown later in the paper is carried out in the one-dimensional meridian direction. The bump curvature is proportional to the curvature of the power density distribution when omitting the heat flux along the length direction of the mirror, and can be written as

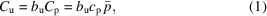




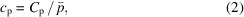

where *C*_p_ is the curvature of the power density distribution, *c*_p_ is the normalized power density distribution curvature, 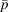 = 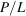 is the average linear power density, *P* is the total power and *L* is the footprint length. *b*_u_ is a scale factor that is irrelevant to *D*.

*D* affects the temperature gradient along the thickness direction of the mirror. The curvature of the bending effect *C*_e_ is proportional to the temperature gradient, which is proportional to 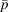 and can be written as

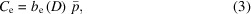

where *b*_e_(*D*) denotes the scale factor relevant to *D*. According to (1) and (3), the total deformation curvature of the WBM can be written as

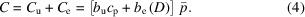

The normalized power density distribution curvature *c*_p_ and the average linear power density 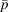 are related to the photon energy regulated by the undulator, and can be written as *c*_p_(*E*) and 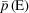 .

This shows that the total thermal deformation curvature *C* is proportional to the total power when the shape of the power density distribution is given. The thermal deformation can be cancelled when *b*_e_ is equal to −*b*_u_ *c*_p_, so *D* is only related to the normalized power density distribution curvature and not the total power.

At a given *D*, the mean-square value of the thermal deformation curvature of each photon energy can be written as

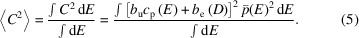

The purpose of optimization over the entire energy range is to find the value of *D* that corresponds to the minimum 〈*C* ^2^〉. As 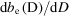 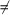 0, we can write 

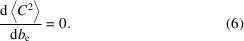

Equation (7) for *b*_e_, which can be written as equation (8) when the photon energy is discrete, is given as follows,

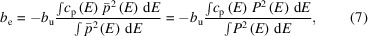




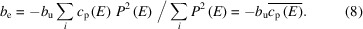

So we can conclude that *D*_opt-all_ can be regarded as *D*_opt_ for 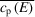 . 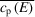 is the average value of the normalized power density distribution curvature weighted by the square of the total power over the whole photon energy range,

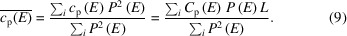

The normalized power density distribution and *D*_opt_ remain unchanged when the power density distribution is multiplied by a constant. Therefore, the power density distribution for *D*_opt-all_ can be replaced by

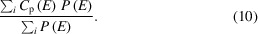

So we can first average the power density distribution weighted by the total power over the entire energy range. Then calculate *D*_opt_ by finite-element analysis (FEA) with this average power density distribution, for which the optimized notch depth can be obtained for the entire energy range (*D*_opt-all_). Thus this approach can drastically reduce the amount of computation with only one round of calculations with FEA.

After obtaining *D*_opt-all_, it is also necessary to check the thermal deformation at each photon energy. We need to find the working status point with the worst thermal deformation quickly. According to equation (4) the thermal deformation curvature of a given photon energy can be written as

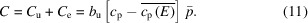

This shows that the worst thermal deformation corresponds to the maximum value that is calculated by multiplying the difference between the normalized power density distribution curvature *c*_p_ and the average value of the normalized power density distribution curvature 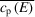 by the average power density (or total power) of the photon energy. It should be noted that there will be a deviation between the absolute curvature of thermal deformation and the slope error (RMS) when there are high-order terms in the power density distribution, which dates from a shortage of smart-cut structure. When the power density distribution has only a quadratic term, the absolute curvature of thermal deformation will be consistent with the slope error (RMS).

## 3. FEA verification

### 3.1. Heat load and cooling structure

The WBM for the HEPS Hard X-ray Imaging beamline is located at *d*_0_ = 37.65 m from the undulator exit end. The grazing angle has been set as θ = 1.7 mrad. The beam footprint size is 553.7 mm × 0.94 mm. The material of the mirror is single-crystal silicon, and the reflective layer includes Rh, Pt and Si. At this grazing-incidence angle and photon energy range (4.8–45 keV), we consider the heat load absorbed by the mirror as the surface power distribution. The curvature of the thermal deformation height value in the effective footprint length in the meridional direction is used to evaluate the thermal deformation of the WBM. This study does not discuss the thermal deformation in the sagittal direction.

**Figure 1:**
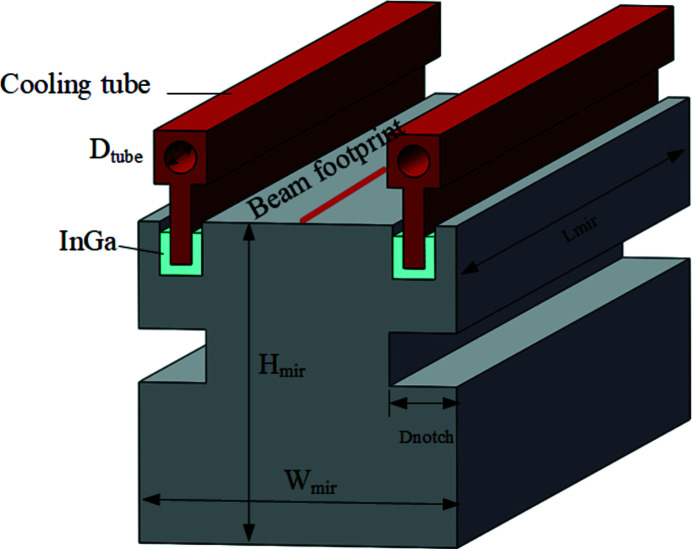
Schematic diagram of the cooling structure, including single-crystal silicon substrate, copper tube and In–Ga bath.

This study adopts the In–Ga bath and water-cooling method. The In–Ga bath is widely used in the synchrotron radiation field because of its good wettability and no clamping force (Vannoni & Freijo-Martín, 2017 ▸; Hardin *et al.*, 2018 ▸; Kitajima *et al.*, 1992 ▸; Ohashi *et al.*, 2004 ▸; Zhang *et al.*, 2017 ▸). The cooling structure is shown in Fig. 1 ▸, with a top-side cooling arrangement. In order to simplify the model, the groove for storing the In–Ga eutectic is not closed, and the length of the mirror is equal to the whole footprint length (WFL). In order to remove the influence of edge effects of the thermal deformation, the size of the beam received by the white-light mirror is larger than the size of the central cone of the beam we want to use. The edge part of the beam is removed by a slit placed behind the WBM, so the length of the central cone size on the mirror surface, the available footprint length (AFL), is 18/25 of WFL (WFL = 554 mm, AFL = 399 mm). The mirror is 554 mm long, and 60 mm wide and thick. The diameter of the cooling tube is 6 mm; the inlet temperature is 25°C; the convective heat transfer boundary condition is 5000 W m^−2^ K^−1^. The notch is 20 mm away from the optical surface of the mirror. Its width is 10 mm, and *D* is the variate to be optimized.

### 3.2. Optimization of the notch

Use of a mirror substrate with notches to control thermal deformation has become a consensus (Khounsary *et al.*, 1999 ▸, 2004 ▸; Zhang *et al.*, 2013 ▸; Brumund *et al.*, 2021 ▸). *D* is a key factor to optimize the mirror substrate which can prevent the bottom of the mirror from becoming too cold.

**Figure 2:**
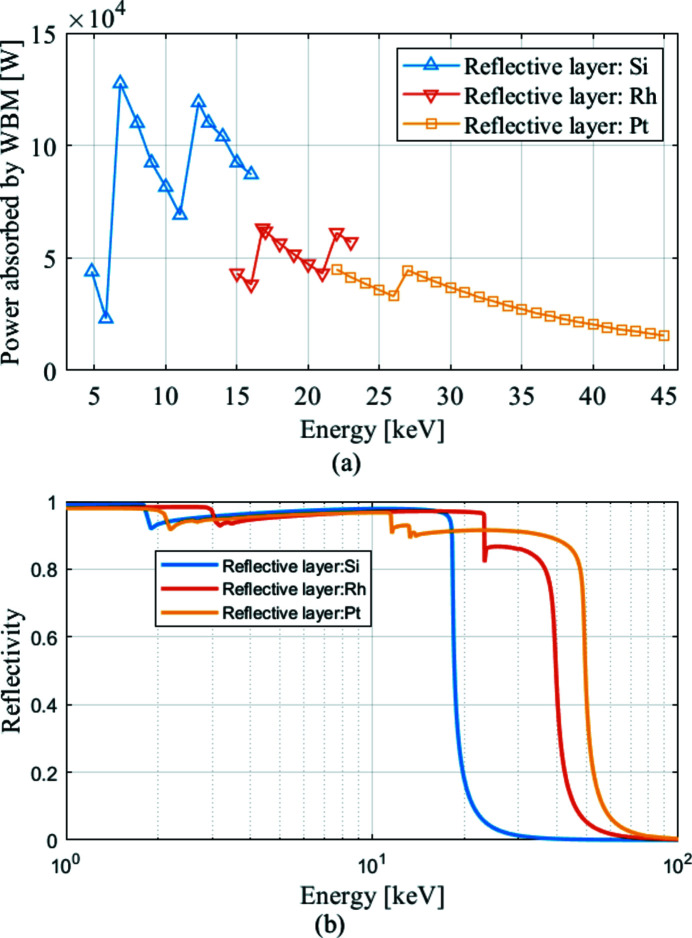
(*a*) Power absorbed by the WBM at different photon energies. The range of the fundamental harmonic is from 4.8 keV to 6.8 keV, the range of the third harmonic is from 6.8 keV to 11 keV, the range of the fifth harmonic is from 12.3 keV to 26 keV, and the range of the seventh harmonic is from 27 keV to 45 keV. (*b*) Reflectivity curve of the Si, Rh and Pt layers.

**Figure 3:**
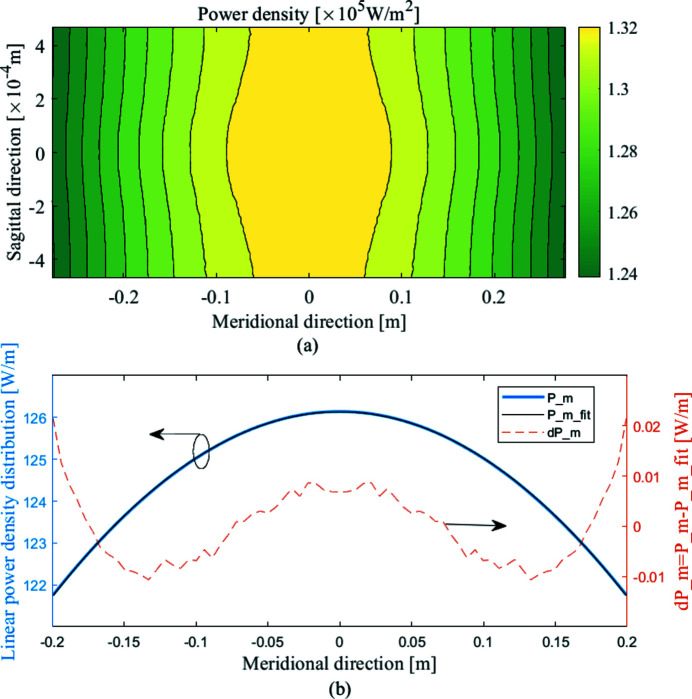
(*a*) Power density distribution for optimization, corresponding to 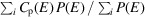 . The normalized power density distribution curvature of the power density distribution for optimization is equal to *c*_p_(*E*). Since all the given power density distribution curvatures are negative, the curvature of the power density distribution for optimization is negative. (*b*) Linear power density of the distribution in the meridian direction.

As shown in Fig. 2 ▸, the power density distribution absorbed by the WBM is calculated for each photon energy using *SPECTRA* (Tanaka & Kitamura, 2001 ▸). The total power ranges from 15.47 W to 127.59 W. The curvatures of the power density distribution for every photon energy are negative, which means the heat load is concentrated in the middle part of the mirror. This paper uses a script program to calculate the power density distribution for optimization based on the theory introduced in Section 2. As shown in Fig. 3 ▸, the total power and the average power density of the power density distribution for optimization are 67.58 W and 0.13 W mm^−2^, respectively. The size of the power density matrix is 101×101 which combined with meshing of the models has to be dense enough to capture the spatial profile of the thermal load in order to correctly compute the temperature distribution. The linear power density of the distribution in the meridian direction (P_m) is shown in Fig. 3 ▸(*b*). Then a best spherical fit is made, to obtain a function P_m_fit. The curvature (*C*_p_) is −221.1 W m^−3^, and the normalized curvature (*c*_p_) is −1.79 m^−2^. In order to show the difference between P_m and P_m_fit, we subtract P_m_fit from P_m. There are still high-order residual terms in the linear power distribution.

**Figure 4:**
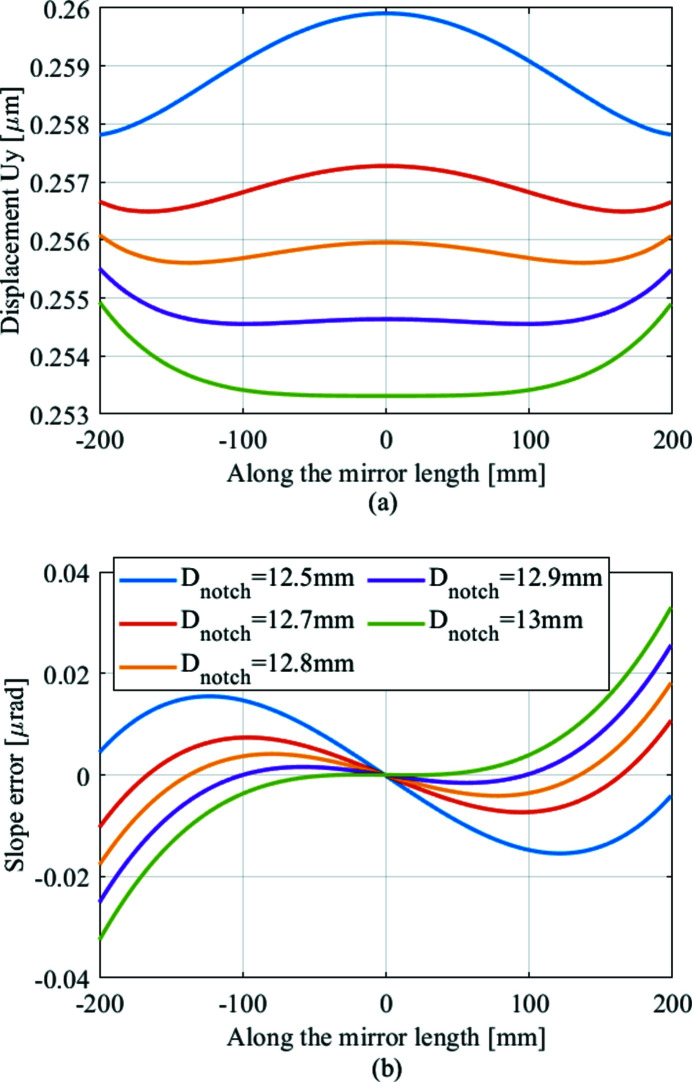
Result of the meridional thermal deformation for different *D*. Displacement *U*_*y*_ (*a*) and slope error (*b*) along the meridian of the footprint are plotted. As *D* increases, thermal deformation changes from convex to concave.

We optimize *D* of the WBM by FEA using the power density distribution for optimization as the input heat load. As shown in Fig. 4 ▸, we obtain the thermal distortion displacement and slope error with different *D* along the mirror meridional direction in the AFL area. The corresponding curvatures and curvature radius are shown in Table 1 ▸. Due to the optimization step size, the curvature of thermal deformation in the AFL area in the meridional direction is optimized to *c*_0_ = −6.03 × 10^−9^ m^−1^ (*c*_0_ is the curvature of thermal deformation of the power density distribution for optimization; by refining the optimization step size, we can make *c*_0_ infinitely close to 0). *D*_opt_ is 12.8 mm, with 5.8 nrad slope error (RMS) and −165700 km curvature radius. Considering the machining tolerance, *D*_opt-all_ can be regarded as 12.8 mm.

## 4. Verification over the entire photon energy range

**Figure 5:**
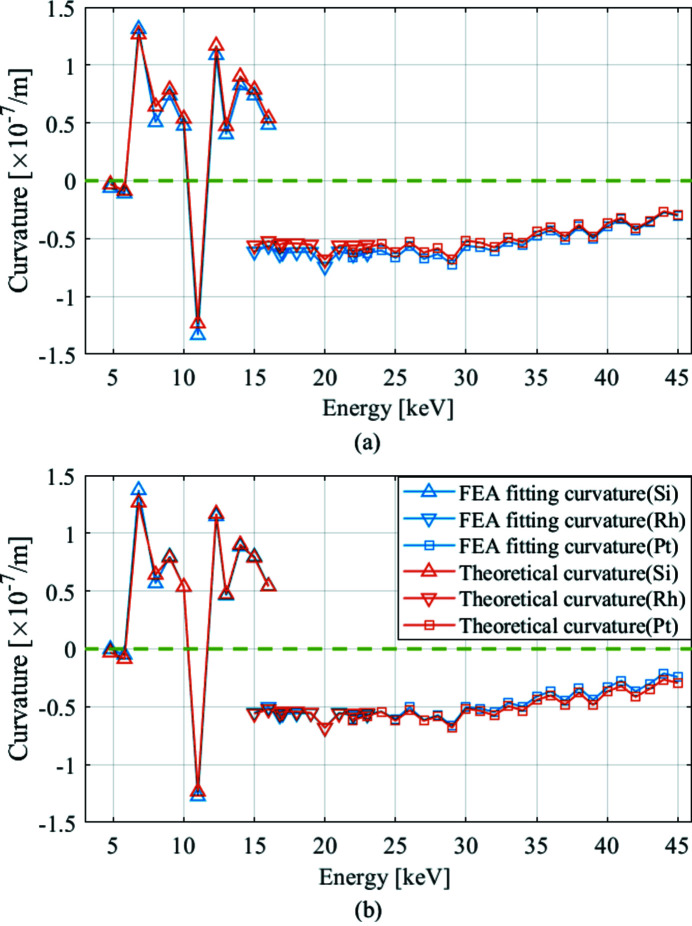
Comparison between theoretical curvatures and FEA fitting curvatures. The green dashed line shows where the curvature equals zero. (*a*) FEA fitting curvatures without calculating *c*_0_ and (*b*) taking *c*_0_ into account.

We calculate the thermal deformation of the WBM over the entire photon energy range by FEA, and obtain the curvatures of the thermal deformation by fitting. We can calculate *b*_u_ in equation (1) using the ratio of FEA curvatures and 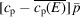 . The value of *b*_u_ in this study is found to be 1.6 × 10^−9^ m^2^ W^−1^. As shown in Fig. 5 ▸(*a*), we obtain the theoretical curvature after considering *b*_u_. There is still a small deviation between the theoretical curvatures and FEA fitting curvatures. The deviation is approximately equal to *C*_0_. Fig. 5 ▸(*b*) shows the result of subtracting *C*_0_ from the FEA fitting curvatures. The theoretical curvatures and FEA fitting curvatures are reasonably well matched both in terms of the positive and negative parts of the curvatures and general trends. This proves the validity of the theory. The positive and negative parts of the curvatures represent concave and convex thermal deformation, respectively. The absolute value of the curvature represents the degree of concave/convex deformation. The larger the absolute curvature, the larger the degree of deformation. When the photon energy is 6.8 keV, the thermal deformation curvature is largest with a 22.2 nrad slope error (RMS). During the design of the WBM, the worst thermal deformation energy point must be taken into account.

## 5. Conclusion

For heat loads generated by synchrotron radiation, it is a challenge to optimize the thermal deformation of mirrors over the entire photon energy range. The thermal deformation optimization theory proposed in this paper requires only one round of optimization calculations, and computation check by FEA. This can significantly reduce the workload of mirror design. Since the power density distribution for optimization is the normalized value of all photon energy points, the *D*_opt-all_ value can ensure that the RMS of the curvatures of the entire photon energy range is minimized; this means our work has taken all the photon energy points into account. Optimizing mirrors at a given energy point should be avoided as it results in large deformations at other energy points. In addition, designers can predict the thermal deformation of a mirror at a given energy point without FEA simulation. This will provide guidance for the correction of the spherical item of the WBM’s thermal deformation by downstream optics, such as focusing mirror (Knopp *et al.*, 2019 ▸), compound refractive lens (CRL) and so on.

## Figures and Tables

**Table 1:** Curvature and curvature radius for different values of *D* Combined with Fig. 4 ▸, negative curvature represents convex thermal deformation, positive curvature represents concave thermal deformation.

*D* (mm)	12.5	12.7	12.8	12.9	13.0
Curvature (×10^−7^ m^−1^)	−1.2	−0.4	−0.06	0.3	0.7
Curvature radius (×10^3^ km)	−8.5	−23.1	−165.7	32.1	14.6
